# Supplementary material for: Getting More Out of Biomedical Documents with GATE's Full Lifecycle Open Source Text Analytics
Source: PLoS Comput Biol. 2013 Feb 7;9(2):e1002854. doi: 10.1371/journal.pcbi.1002854 (PMC3567135; doi:10.1371/journal.pcbi.1002854)
Supplement: Dataset S1 — GATE software. Dataset S1 bundled with this paper contains a distribution of GATE (or see http://gate.ac.uk/download/). (TGZ) [file pcbi.1002854.s001.tgz › plos-gate/plugins/Gazetteer_LKB/doc/team-list.html]

The Large KB gazetteer - Team list


---

Last Published: 2009-10-17

---

##### Large KB Gazetteer

- Overview
- Usage
- Q & A
- GATE Compatibility
- GATE Gazetteer Feature Comparison
- Semantic Enrichment PR
- Samples

##### Developer Documentation

- Dictionary Lifecycle

##### Project Documentation

- Project Information
  - Continuous Integration
  - Dependencies
  - Dependency Management
  - Issue Tracking
  - Mailing Lists
  - Plugin Management
  - Project License
  - Project Plugins
  - Project Summary
  - **Project Team**
  - Source Repository
- Project Reports

## The Team

A successful project requires many people to play many roles. Some members write code or documentation, while others are valuable as testers, submitting patches and suggestions.

The team is comprised of Members and Contributors. Members have direct access to the source of a project and actively evolve the code-base. Contributors improve the project through submission of patches and suggestions to the Members. The number of Contributors to the project is unbounded. Get involved today. All contributions to the project are greatly appreciated.

### Members

The following is a list of developers with commit privileges that have directly contributed to the project in one way or another.

| Id | Name | Email | Roles |
| --- | --- | --- | --- |
| danko | Danail Kozhuharov | danko@ontotext.com | developer |
| mnozhchev | Marin Nozhchev | marin.nozhchev@ontotext.com | lead developer |
| reni | Reneta Popova | reneta.popova@ontotext.com | technical writer |

### Contributors

There are no contributors listed for this project. Please check back again later.

---

©
2009
Ontotext AD

---
